# Accelerating calculations of RNA secondary structure partition functions using GPUs

**DOI:** 10.1186/1748-7188-8-29

**Published:** 2013-11-01

**Authors:** Harry A Stern, David H Mathews

**Affiliations:** 1 Center for Integrated Research Computing, Taylor Hall, University of Rochester, Rochester, NY, 14627, USA; 2 Department of Biochemistry & Biophysics and Center for RNA Biology, University of Rochester Medical Center, 601, Elmwood Avenue Box 712, Rochester, NY, 14642, USA

**Keywords:** RNA, Secondary structure, Partition function, Graphical processing unit, CUDA

## Abstract

**Background:**

RNA performs many diverse functions in the cell in addition to its role as a messenger of genetic information. These functions depend on its ability to fold to a unique three-dimensional structure determined by the sequence. The conformation of RNA is in part determined by its secondary structure, or the particular set of contacts between pairs of complementary bases. Prediction of the secondary structure of RNA from its sequence is therefore of great interest, but can be computationally expensive. In this work we accelerate computations of base-pair probababilities using parallel graphics processing units (GPUs).

**Results:**

Calculation of the probabilities of base pairs in RNA secondary structures using nearest-neighbor standard free energy change parameters has been implemented using CUDA to run on hardware with multiprocessor GPUs. A modified set of recursions was introduced, which reduces memory usage by about 25%. GPUs are fastest in single precision, and for some hardware, restricted to single precision. This may introduce significant roundoff error. However, deviations in base-pair probabilities calculated using single precision were found to be negligible compared to those resulting from shifting the nearest-neighbor parameters by a random amount of magnitude similar to their experimental uncertainties. For large sequences running on our particular hardware, the GPU implementation reduces execution time by a factor of close to 60 compared with an optimized serial implementation, and by a factor of 116 compared with the original code.

**Conclusions:**

Using GPUs can greatly accelerate computation of RNA secondary structure partition functions, allowing calculation of base-pair probabilities for large sequences in a reasonable amount of time, with a negligible compromise in accuracy due to working in single precision. The source code is integrated into the RNAstructure software package and available for download at http://rna.urmc.rochester.edu.

## Background

RNA performs many diverse functions in the cell in addition to its role as a messenger of genetic information. It can form enzymes, for example for cleavage of itself or of other RNA, or to create peptide bonds as a fundamental constituent of the ribosome [1]. It can act as a signalling molecule for regulation of gene expression, for protein export, or for guiding post-translational modifications [2-5].

As for proteins, RNA function depends on its folding to a well-defined three-dimensional shape. In contrast to proteins, the folding of RNA is hierarchical [6]. Secondary structure, or the particular set of contacts between pairs of complementary bases mediated by hydrogen bonding and stacking of bases, provides a significant amount of information. This can be helpful in predicting function or accessibility to ligands [7-10]. Computational prediction of the secondary structure of RNA from its sequence is therefore of great interest. The most widely-used automated prediction methods attempt to estimate the thermodynamic stability of RNA, using empirical parameters determined from experiments on oligonucleotides [11,12].

CUDA is a programming interface developed by the company NVIDIA to facilitate general-purpose, parallel high-performance computing on multiprocessor graphics processing units (GPUs) [13]. In recent years, many scientific computing applications have been implemented on GPUs using CUDA, in many cases yielding speed-ups of several orders of magnitude [14,15]. However, to our knowledge, only a handful of publications have appeared describing GPU implementations of codes for RNA secondary structure prediction. Rizk and Lavenier described a CUDA implementation of structure prediction by free energy minimization [16]. Their work was limited to relatively short sequences (up to 120 bases) and a simplified energy model that neglects coaxial stacking. The GPU implementation was faster than the serial implementation by a factor of 10 and 17 depending on the particular hardware. More recently, Lei et al. [17] also reported a parallelized implementation of free energy minimization using CUDA. They used only a coarse-grained parallelization scheme where the minimum-free-energy structures for subsequences of a given length are calculated in parallel, but the search over structures for each subsequence is done serially. Their work was also limited to relatively short sequences (up to 221 bases). They reported speedups of up to a factor of 16. It should be noted that these parallelized implementations neither use the latest thermodynamic parameters for loops [18], nor include coaxial stacking interactions [19].

In addition, recent work demonstrates that calculations of base-pairing probabilities calculated with partition functions can provide additional useful information [20]. Structures composed of highly probable pairs are more accurate than lowest free energy structures [20,21]. The base pair probabilities provide confidence intervals for prediction of individual pairs. Base pairing probabilities can also be used to predict pseudoknots [22]. We have additionally extended this work to predictions using multiple, homologous sequences, where the same principles hold true [23-26].

In this paper, we present the calculation of base-pair probabilities for 44 sequences containing up to 10,000 bases, using an optimized and parallelized version of the “partition” code in the RNAstructure package [27]. Our test set contained both random sequences of varying lengths (up to 10,000 bases) and actual sequences of biological importance (see Table 1), the longest being the HIV-1 NL43 genome (GenBank: AF324493) containing 9709 bases.

**Table 1:** Biological RNA sequences examined^*^

**Sequence**	**# bases**	**Reference**
tRNA RQ2640	75	[28]
tRNA RD0500	76	[28]
tRNA RA7680	76	[28]
tRNA RD0260	77	[28]
tRNA RR1664	77	[28]
*Candida albicans* 5S rRNA	114	[29]
*Escherichia coli* 5S rRNA	120	[30]
P546 folding domain of *Tetrahymenathermophilia* group I intron	155	[31]
*Bacillus stearothermophilus* SRP RNA	268	[32]
3’ UTR of *Bombyx mori* R2 element with flanking vector sequence	300	[33]
*Tetrahymena thermophilia* group I intron	433	[30]
*Saccharomyces cerevisiae* A5 group II intron	632	[34]
*Escherichia coli* small subunit rRNA	1542	[30]
*Escherichia coli* large subunit rRNA	2904	[29]
human ICAM-1 mRNA	2986	[35]
HIV-1 NL43 genome (GenBank: AF324493)	9709	[36]

^*^28 random sequences of length 10–10,000 were also examined.

We employed a sophisticated and accurate energy calculation that includes coaxial stacking [19]. Before attempting a parallel implementation, we first wrote an optimized serial version of the original code (the “partition” program in RNAstructure), implementing only a subset of its functionality, while improving efficiency and reducing memory usage. Subsequently, we parallelized the optimized code for GPU hardware, using CUDA. Here we made use of a fine-grained parallelization scheme, in which the calculation of the restricted partition function for each subsequence of a given length is parallelized, as well as the calculation of the restricted partition function for each subsequence. We found that this fine-grained parallelization resulted in greater speedups than a simpler coarse-grained-only parallelization (up to factors of ∼60 compared to the optimized serial version and ∼116 compared to the original code).

## Implementation

### Calculating base pair probabilities

The probability *p*_*i*,*j*_ of a canonical pair between bases *i* and *j* related to the standard free energy change $\Delta G_{i,j}^{0}$ of the restricted ensemble of structures containing the pair, and the standard free energy change *ΔG*^0^ of the unrestricted ensemble of all possible structures, both relative to the state in which all bases are unpaired:

(1) $$p_{i,j}=\frac{w\left(\Delta G_{i,j}^{0}\right)}{w\left(\Delta G^{0}\right)}$$

where

(2) $$w(g)\equiv \;\exp\;\left(-g/\text{RT}\right)$$

is the Boltzmann weight corresponding to standard free energy change *g* at temperature *T*.

The standard free energy changes $\Delta G_{i,j}^{0}$ and *ΔG*^0^ are estimated using the Turner nearest-neighbor rules [11,18,37], and pseudoknots are excluded. In that case, $\Delta G_{i,j}^{0}$ depends on two independent contributions, one for the interior fragment containing the pair *i*,*j* and bases in between (but excluding bases from the 5’ end to *i*−1 and from *j*+1 to the 3’ end), and one for the exterior fragment containing the pair and bases from the two ends (but excluding bases from *i*+1 to *j*−1). We define *V*_*i*,*j*_ to be the relative standard free energy change for the interior fragment in the case that *i*<*j* and for the exterior fragment otherwise, following the convention used in the mfold prediction software [38]. In that case,

(3) $$\Delta G_{i,j}^{0}=-\text{RT}\log\left[\exp\left(-V_{i,j}/\text{RT}\right)+\exp\left(-V_{j,i}/\text{RT}\right)\right]$$

or more succinctly

(4) $$w\left(\Delta G_{i,j}^{0}\right)=w\left(V_{i,j}\right)+w\left(V_{j,i}\right)$$

The standard free energy changes *V*_*i*,*j*_ are calculated using the following set of recursions:

(5) $$w\left(V_{i,j}\right)=\left\{\begin{array}{ll} Q_{i,j}^{\text{hairpin}}+Q_{i,j}^{\text{stack}}+Q_{i,j}^{\text{internal}}+Q_{i,j}^{\text{multibranch}} & \;\text{for}\;i<j\\ Q_{i,j}^{\text{exterior}}+Q_{i,j}^{\text{stack}}+Q_{i,j}^{\text{internal}}+Q_{i,j}^{\text{multibranch}} & \;\text{for}\;i>j \end{array}\right)$$

where

(6) $$\begin{array}{l} Q_{i,j}^{\text{hairpin}}\;=\;w\left(\Delta G_{i,j}^{\text{hairpin}}\right) \end{array}$$

(7) $$\begin{array}{l} Q_{i,j}^{\text{stack}}\;=\;w\left(V_{i+1,j-1}+\Delta G_{i,j,i+1,j-1}^{\text{stack}}\right) \end{array}$$

(8) $$\begin{array}{l} Q_{i,j}^{\text{internal}}\;=\;\underset{i<i^{\prime }<j^{\prime }<j}{\sum }w\left(V_{i^{\prime },j^{\prime }}+\Delta G_{i,j,i^{\prime },j^{\prime }}^{\text{internal}}\right) \end{array}$$

(9) $$\begin{array}{l} Q_{i,j}^{\text{multibranch}}\;=\;w\left(W_{i+1,j-1}^{\text{MB}}+a+c\right)+w\left(W_{i+2,j-1}^{\text{MB}}+\Delta G_{i,j,i+1}^{3^{\prime }\text{dangle}}+a+b+c\right)+\\ \;w\left(W_{i+1,j-2}^{\text{MB}}+\Delta G_{i,j,j-1}^{5^{\prime }\text{dangle}}+a+b+c\right)+\\ \;w\left(W_{i+2,j-2}^{\text{MB}}+\Delta G_{i,j,i+1,j-1}^{\mathrm{terminal}\;\text{mismatch}}+a+2b+c\right)+\\ \;\underset{i<k<j}{\sum }w\left(V_{i+1,k}+Y_{k+1,j-1}+\Delta G_{j,i,i+1,k}^{\text{coaxial flush}}+a+2c\right)+\\ \;\underset{i<k<j}{\sum }w\left(V_{i+2,k}+Y_{k+2,j-1}+\Delta G_{j,i,i+2,k}^{\text{coaxial mismatch(2)}}+a+2b+2c\right)+\\ \;\underset{i<k<j}{\sum }w\left(V_{i+2,k}+Y_{k+1,j-2}+\Delta G_{j,i,i+2,k}^{\text{coaxial mismatch(1)}}+a+2b+2c\right)+\\ \;\underset{i<k<j}{\sum }w\left(V_{k,j-1}+Y_{i+1,k-1}+\Delta G_{k,j-1,j,i}^{\text{coaxial flush}}+a+2c\right)+\\ \;\underset{i<k<j}{\sum }w\left(V_{k,j-2}+Y_{i+1,k-2}+\Delta G_{k,j-2,j,i}^{\text{coaxial mismatch(1)}}+a+2b+2c\right)+\\ \;\underset{i<k<j}{\sum }w\left(V_{k,j-2}+Y_{i+2,k-1}+\Delta G_{k,j-2,j,i}^{\text{coaxial mismatch(2)}}+a+2b+2c\right) \end{array}$$

(10) $$\begin{array}{l} Q_{i,j}^{\text{exterior}}\;=\;w\left(W_{i+1}^{3^{\prime }}+W_{j-1}^{5^{\prime }}\right)+w\left(W_{i+2}^{3^{\prime }}+W_{j-1}^{5^{\prime }}+\Delta G_{i,j,i+1}^{3^{\prime }\text{dangle}}+\right)+\\ \;w\left(W_{i+1}^{3^{\prime }}+W_{j-2}^{5^{\prime }}+\Delta G_{i,j,j-1}^{5^{\prime }\text{dangle}}+\right)+w\left(W_{i+2}^{3^{\prime }}+W_{j-2}^{5^{\prime }}+\Delta G_{i,j,i+1,j-1}^{\mathrm{terminal}\;\text{mismatch}}\right)+\\ \;\underset{i<k<j}{\sum }w\left(V_{i+1,k}+W_{k+1}^{3^{\prime }}+W_{j-1}^{5^{\prime }}+\Delta G_{j,i,i+1,k}^{\text{coaxial flush}}\right)+\\ \;\underset{i<k<j}{\sum }w\left(V_{i+2,k}+W_{k+2}^{3^{\prime }}+W_{j-1}^{5^{\prime }}+\Delta G_{j,i,i+2,k}^{\text{coaxial mismatch(2)}}\right)+\\ \;\underset{i<k<j}{\sum }w\left(V_{i+2,k}+W_{k+1}^{3^{\prime }}+W_{j-2}^{5^{\prime }}+\Delta G_{j,i,i+2,k}^{\text{coaxial mismatch(1)}}\right)+\\ \;\underset{i<k<j}{\sum }w\left(V_{k,j-1}+W_{i+1}^{3^{\prime }}+W_{k-1}^{5^{\prime }}+\Delta G_{k,j-1,j,i}^{\text{coaxial flush}}\right)+\\ \;\underset{i<k<j}{\sum }w\left(V_{k,j-2}+W_{i+1}^{3^{\prime }}+W_{k-2}^{5^{\prime }}+\Delta G_{k,j-2,j,i}^{\text{coaxial mismatch(1)}}\right)+\\ \;\underset{i<k<j}{\sum }w\left(V_{k,j-2}+W_{i+2}^{3^{\prime }}+W_{k-1}^{5^{\prime }}+\Delta G_{k,j-2,j,i}^{\text{coaxial mismatch(2)}}\right) \end{array}$$

(11) $$\begin{array}{l} w\left(W_{i,j}^{L}\right)\;=\;w\left(W_{i+1,j}^{L}+b\right)+w\left(V_{i,j}+c\right)+w\left(V_{i,j-1}+\Delta G_{j-1,i,j}^{3^{\prime }\text{dangle}}+b+c\right)+\\ \;w\left(V_{i+1,j}+\Delta G_{j,i+1,i}^{5^{\prime }\text{dangle}}+b+c\right)+w\left(V_{i+1,j-1}+\Delta G_{j-1,i+1,j,i}^{\mathrm{terminal}\;\text{mismatch}}+2b+c\right) \end{array}$$

(12) $$\begin{array}{l} w\left(W_{i,j}^{Q}\right)\;=\;w\left(V_{i,j}\right)+w\left(V_{i,j-1}+\Delta G_{j-1,i,j}^{3^{\prime }\text{dangle}}\right)+w\left(V_{i+1,j}+\Delta G_{j,i+1,i}^{5^{\prime }\text{dangle}}\right)+\\ \;w\left(V_{i+1,j-1}+\Delta G_{j-1,i+1,j,i}^{\mathrm{terminal}\;\text{mismatch}}\right) \end{array}$$

(13) $$\begin{array}{l} w\left(W_{i,j}\right)\;=\;w\left(W_{i,j-1}+b\right)+w\left(W_{i,j}^{L}\right) \end{array}$$

(14) $$\begin{array}{l} w\left(W_{i,j}^{\text{coax}}\right)\;=\;\underset{i<k<j}{\sum }w\left(V_{i,k}+V_{k+1,j}+\Delta G_{i,k,k+1,j}^{\text{coaxial flush}}+2c\right)+\\ \;\underset{i<k<j}{\sum }w\left(V_{i+1,k}+V_{k+2,j}+\Delta G_{i+1,k,\text{kj}+2,j}^{\text{coaxial mismatch(1)}}+2b+2c\right)+\\ \;\underset{i<k<j}{\sum }w\left(V_{i,k}+V_{k+2,j-1}+\Delta G_{i,k,k+2,j-1}^{\text{coaxial mismatch(2)}}+2b+2c\right) \end{array}$$

(15) $$\begin{array}{l} w\left(Z_{i,j}\right)\;=\;w\left(W_{i,j}^{\text{coax}}\right)+w\left(V_{i,j}+c\right)+w\left(V_{i,j-1}+\Delta G_{j-1,i,j}^{3^{\prime }\text{dangle}}+b+c\right)+\\ \;w\left(V_{i+1,j}+\Delta G_{j,i+1,i}^{5^{\prime }\text{dangle}}+b+c\right)+w\left(V_{i+1,j-1}+\Delta G_{j-1,i+1,j,i}^{\mathrm{terminal}\;\text{mismatch}}+2b+c\right) \end{array}$$

(16) $$\begin{array}{l} w\left(W_{i,j}^{\text{MBL}}\right)\;=\;w\left(W_{i+1,j}^{\text{MBL}}\right)+w\left(W_{i,j}^{\text{coax}}\right)+\underset{i<k<j}{\sum }w\left(Z_{i,k}+Y_{k+1,j}^{L}\right) \end{array}$$

(17) $$\begin{array}{l} w\left(W_{i,j}^{\text{MB}}\right)\;=\;w\left(W_{i,j-1}^{\text{MB}}+b\right)+w\left(W_{i,j}^{\text{MBL}}\right) \end{array}$$

(18) $$\begin{array}{l} w\left(Y_{i,j}\right)\;=\;w\left(W_{i,j}\right)+w\left(W_{i,j}^{\text{MB}}\right) \end{array}$$

(19) $$\begin{array}{l} w\left(Y_{i,j}^{L}\right)\;=\;w\left(W_{i,j}^{L}\right)+w\left(W_{i,j}^{\text{MBL}}\right) \end{array}$$

(20) $$\begin{array}{l} w\left(W_{i}^{5^{\prime }}\right)\;=\;w\left(W_{i-1}^{5^{\prime }}\right)+\underset{j<i}{\sum }w\left(W_{j-1}^{5^{\prime }}+W_{j,i}^{Q}\right) \end{array}$$

(21) $$\begin{array}{l} w\left(W_{i}^{3^{\prime }}\right)\;=\;w\left(W_{i+1}^{3^{\prime }}\right)+\underset{j>i}{\sum }w\left(W_{j+1}^{3^{\prime }}+W_{i,j}^{Q}\right) \end{array}$$

These recursions are slightly different from—but equivalent to—those presented in reference [20] and used in the previous code. It should be noted that there was an error in equation 15 of reference [20]: in the second line, WMBL(k+1,j) should be replaced by [WMBL(k+1,j) + WL(k+1,j)].

The quantities *V*, *W*, *W*^MB^, *W*^L^, *W*^coax^, *W*^5′^, and *W*^3′^ are simply −*RT* times the logarithms of the quantities in reference [20]. In addition, four new arrays are introduced: 

1. *W*^Q^ is the standard free energy change corresponding to the sum of terms on the right hand side of equation 11 of reference [20] not including the scaling by W5(k).

2. Elements of *Y* are −*RT* times the logarithm of the sum of the Boltzmann weights of corresponding elements of *W* and *W*^MB^.

3. Elements of *Y*^L^ are −*RT* times the logarithm of the sum of the Boltzmann weights of corresponding elements of *W*^L^ and *W*^MBL^.

4. Elements of *Z* are −*RT* times the logarithm of the sum of the Boltzmann weights of corresponding elements of *W*^L^ (except for the term depending on the next-smallest fragment) and *W*^coax^.

Reorganizing the recursions in this way might appear to use more memory because of the additional arrays. In fact, the modified version requires less memory, because several of the arrays do not need to be stored in their entirety. Specifically, using the modified recursions, storage is only required for two diagonals of *W*, *W*^L^, and *W*^MBL^; for five diagonals of *W*^MB^; and for a half-triangle of *W*^Q^. Reducing memory usage is important as the size of the full arrays scales as *O*(*N*^2^) and the available GPU memory on our hardware was limited to ∼2.5 GB. The modified recursions use four full *N*×*N* arrays and one half-triangle, rather than the six full *N*×*N* arrays used in the original recursions, and therefore reduce memory usage by about 25%. In addition, the calculation of *W*^5′^ and *W*^3′^ is simplified (compare equations 20 and 21 above with equation 11 of reference [20]).

As in the previous work, the various *ΔG* parameters above are from the Turner nearest-neighbor rules [11], while *a*,*b*, and *c* are from the following estimate of the standard free energy change for multibranch loop initiation:

(22) $$\Delta G^{0}=a+\text{bn}+\text{ch}$$

Here *n* is the number of unpaired nucleotides and *h* is the number of branching helices [21]. By convention, the size of internal loops is limited to thirty unpaired nucleotides, so the number of terms in equation 8 and the overall computational expense scales as *O*(*N*^3^) where *N* is the size of the sequence.

The largest difference in the new implementation is that logarithms of probabilities and partition functions (i.e., standard free energy changes) are used rather than probabilities themselves, which is convenient when working in single precision in order to avoid overflow or underflow errors. This requires that exponentials and logarithms are calculated at each step of the calculation where sums are performed. This approach is a departure from the previous implementation, which used scaling factors. Having to compute logarithms and exponentials does entail some additional computational expense, but this does not appear to be exhorbitant on the GPU, because optimized intrinsic mathematical functions are used. (i.e., the code was compiled with the NVIDIA compiler using the -use_fast_math option). The function required for the sum of two free energies *a* and *b* (expressed in units such that *RT*=1) is

(23) $$f(a,b)=-\log\left(e^{-a}+e^{-b}\right)$$

We calculated this in the following way:

(24) $$f(a,b)=\left\{\begin{array}{cc} a-\log\left(1+e^{a-b}\right)\; & a<b\\ b-\log\left(1+e^{b-a}\right)\; & a>b\\ a-\log(2)\; & a=b \end{array}\right)$$

This was done for two reasons: it requires at most a single call to exp, rather than two; and it can make use of the log1p function from the standard math library, which calculates log(1+*x*) accurately even for small *x*. This is important because often *e*^−*a*^ and *e*^−*b*^ will differ by several orders of magnitude, and simply adding them and then taking the logarithm can lead to significant roundoff error.

In order to determine how much additional computational overhead was imposed by the calculation of exp and log1p we performed a comparison with an artificial reference calculation, which was identical except that calls to these functions were omitted. We found that for a 1,000-mer, the actual GPU calculation is only ∼20% more expensive than this reference calculation.

For a serial calculation on the CPU, there is a larger performance hit; the actual calculation is about a factor of two more expensive than the reference without exp or log1p. However, it should be noted that this is not the entire story, because overall, the new optimized serial code, which uses logarithms, is still faster than the original code, which does not. Running the calculation in log space results in simplifications such as not requiring checking for overflow and not having to multiply by scaling factors, which reduces computational expense.

### Parallelization of the partition function calculation using CUDA

In the CUDA programming model, overall execution of the program is still governed by the CPU. Compute-intensive portions of the program are then delegated to subroutines executed by the GPU, or kernels. In general, the GPU has its own memory, so data must be copied to and from the GPU before and after kernel execution. Many copies of a kernel, or threads, run in parallel, each of which belongs to a block. During kernel execution, threads belonging to the same block can share data and synchronize, whereas threads belonging to different blocks cannot. A program can contain many kernels, which can execute either serially or in parallel [13].

The algorithm for calculating partition functions is recursive: partition functions for larger fragments depend on those for smaller fragments. As such, the overall calculation proceeds serially, in order of fragment size. We used two levels of parallelization, a block level and a thread level. Calculations for all fragments of a given size may be done in parallel, with no communication. This was implemented in CUDA at the level of blocks of threads. The partition function for a given fragment depends on sums, with the number of terms on the order of the fragment size (e.g., equation 9). These sums were parallelized at the level of threads within a block, since calculating a sum in parallel relies on communication between the threads. In our experience, a greater speedup was obtained from this “inner loop” parallelization, even though it requires more communication between threads. Most likely, this is because optimal efficiency on GPU hardware is obtained when identical mathematical operations are performed in lockstep on different data [13]. We stress that these two different levels of parallelization are not mutually exclusive and optimal performance was obtained from including both. A separate block of threads was run for each fragment, while 256 threads were run within a block. The number of threads per block was chosen by trial and error and was optimal for our hardware (the simple sum reduction scheme we chose requires it to be a power of two). In our code, this value is set at compile time (but this is not required by CUDA—it could be set at run time if desired).

## Results and discussion

### Accuracy

Peak floating point performance for NVIDIA Tesla GPUs are faster by a factor of two when working in single compared with working in double precision (http://www.nvidia.com), but single precision introduces greater roundoff error. In order to examine accuracy, we calculated base-pair probabilities for the same sequences using both the parallel CUDA/GPU implementation in single precision, and the serial implementation in double precision. We also calculated probabilities in double precision using a set of nearest-neighbor parameters slightly modified by adding a random variate chosen from a Gaussian distribution with mean 0 and standard deviation 0.01 kcal/mol, which is comparable to or smaller than their experimental uncertainty (0.1 kcal/mol for parameters describing helical stacking and 0.5 kcal/mol for parameters describing loops [18,37]).

**Figure 1:**
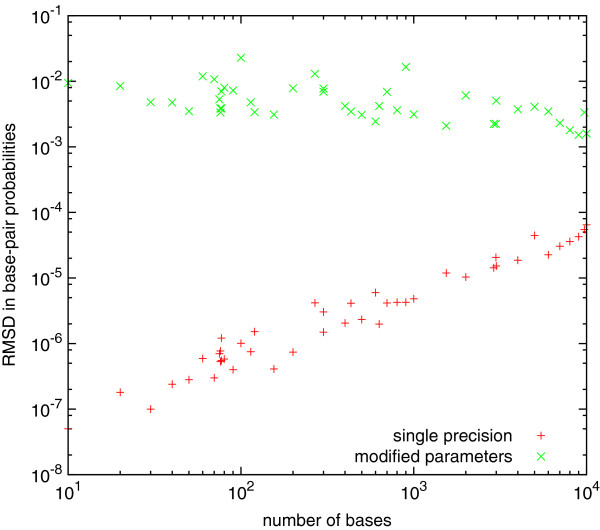
**Accuracy of base-pair probabilities.** Root-mean-square deviation in base-pair probabilities calculated using the CUDA single-precision implementation (red plus signs) and the serial double-precision implementation with slightly modified parameters (green crosses), compared with the serial double-precision implementation using the original parameters.

Figure 1 shows the root-mean-square deviation (RMSD) between base-pair probabilities calculated using double precision and either single precision, or the modified parameters. The RMSD of the calculation using modified parameters decreases as the size of the sequences increases (because the fraction of pairs with very small probabilities increases). In contrast, the roundoff error due to working in single precision increases as the cube of the number of bases in the sequence (i.e., the number of operations involved in the calculation). However, it remains relatively small for sequences of up to 10,000 bases, and negligible compared with the differences resulting from the modified parameters. Our conclusion is that working in single precision does not introduce unacceptable roundoff error for RNA secondary structure prediction for sequences of this size and most likely substantially larger.

We also used the calculated base pair probabilities to determine a single consensus structure for each sequences, using the ProbKnot algorithm [22]. In this case, working in single precision led to differences with double precision only for one sequence (a random sequence of 6000 bases) out of the 44 we examined, and these were very small (only three bases out of the 6000 were matched with a different partner). Working with the modified parameters led to larger discrepancies although these were still fairly small (for sequences containing more than 100 bases, at most 6% of bases were matched with different partners). This is consistent with a previous report that predictions using base pair probabilities are significantly less sensitive to errors in thermodynamic parameters than using only lowest free energy structures [39].

### Computational expense

**Figure 2:**
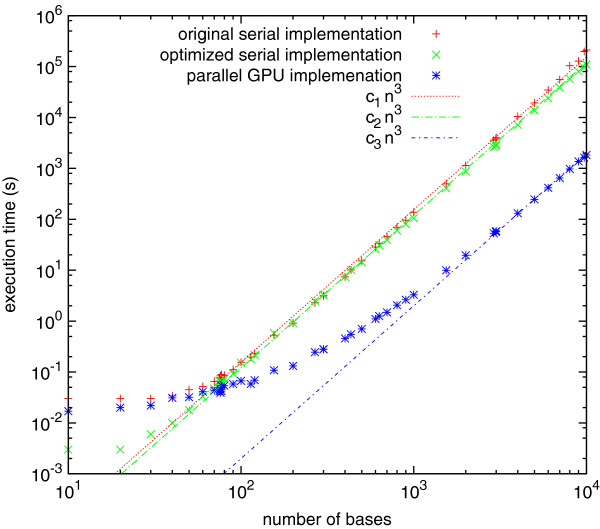
**Computational expense.** Execution time in seconds for the original serial implementation (red plus signs), optimized serial implementation (green crosses), and parallel GPU implementation (blue asterisks). The red, green, and blue lines are the cube of the number of bases *n* in the sequences, multiplied by a constant chosen to best fit the corresponding execution times.

We compared overall execution time for the original serial code developed in our laboratory, the optimized serial code, and the parallel CUDA/GPU implementation. Calculations were performed for sequences containing from 10 to 10,000 bases, on compute nodes containing dual hex-core Intel Xeon 2.67 GHz CPUs and dual NVIDIA Tesla M2050 GPUs, each of which contains 448 multiprocessor cores (only a single GPU was used). Figure 2 shows the execution time (from the user time reported by the UNIX “time” command) of the original and optimized serial codes as well as the parallel CUDA/GPU implementation. The execution time for all codes scales as the cube of the sequence length for large sequences. The CUDA version was able to calculate base-pair probabilities for the sequence for the full HIV-1 NL43 genome (9709 nucleotides) in 27 minutes. We note that there is a small overhead (a few hundredths of a second) involved in running calculations with either the original serial code or the CUDA code, which is not present for the optimized serial code. This overhead has different origins: for the original code, it is probably due to reading the parameter files from disk, while for the CUDA code, it is most likely due to copying parameters and other data between the GPU and CPU.

## Conclusions

In this work, we introduced a modified set of recursions for calculating RNA secondary structure partition functions and base-pairing probabilities using a dynamic programming algorithm, and implemented these in parallel using the CUDA framework for multiprocessor GPUs. For large sequences, the GPU implementation reduces execution time by a factor of close to 60 compared with an optimized serial implementation, and by a factor of 116 compared with the original code. It is clear from our work that using GPUs can greatly accelerate computation of RNA secondary structure partition functions, allowing calculation of base-pair probabilities for large sequences in a reasonable amount of time, with a negligible compromise in accuracy due to working in single precision. It is expected that parallelization using CUDA should be applicable to other implementations of dynamic programming algorithms [12] besides ours, and result in similar speedups.

Two levels of parallelization were implemented. Calculations for all fragments of a given size were done in parallel, with no communication between threads. This was implemented in CUDA at the level of blocks of threads. In addition, the sums contributing to the partition function for a given fragment were calculated in parallel, with communication required between threads. These sums were parallelized at the level of thread within a block. We found that this “inner loop” parallelization resulted in a significantly greater speedup than the “outer loop” parallelization alone.

## Availability and requirements

● Project name: partition-cuda; part of RNAstructure, version 5.5 and later

● Project home page: http://rna.urmc.rochester.edu/RNAstructure.html

● Operating system(s): Unix

● Programming languages: C and CUDA

● Other requirements: CUDA compiler, available from

● http://www.nvidia.com/object/cuda\_home\_new.html

● License: GNU GPL

● Any restrictions to use by non-academics: None.

## Competing interests

The authors declare that they have no competing interests.

## Authors’ contributions

HAS and DHM conceived of this work. HAS implemented the new partition function recursions for serial and parallel execution and performed the test calculations. HAS wrote the first draft of the manuscript, and DHM provided input to the final manuscript. Both authors read and approved the final manuscript.
